# Editorial: Aquatic genomics and transcriptomics for evolutionary biology

**DOI:** 10.3389/fgene.2023.1183637

**Published:** 2023-05-05

**Authors:** Tatiana V. Ovchinnikova, Qiong Shi

**Affiliations:** ^1^ M.M. Shemyakin and Yu.A. Ovchinnikov Institute of Bioorganic Chemistry, Russian Academy of Sciences, Moscow, Russia; ^2^ Department of Bioorganic Chemistry, Faculty of Biology, Lomonosov Moscow State University, Moscow, Russia; ^3^ Shenzhen Key Lab of Marine Genomics, Guangdong Provincial Key Lab of Molecular Breeding in Marine Economic Animals, BGI Academy of Marine Sciences, Shenzhen, China

**Keywords:** genomics, transcriptomics, evolution, aquatic animals, evolutionary biology

Over the last years, plethora of bioactive macromolecules have been isolated from aquatic organisms. Taking in account that more than two-thirds of the global surface area is covered with water, aquatic species constitute above a half of the total biodiversity. Long-term evolution of aquatic animals has led to their irreplaceability in water ecosystems and environments. Many molecular factors such as ancient components of innate immunity system play a key role in host defense of aquatic animals. They are involved in basic mechanisms of survival, growth, reproduction, and homeostasis of living organisms.

Genomics, transcriptomics, and bioinformatics have been rapidly developed for study in the field of aquatic biology due to recent advances in next-generation sequencing techniques and big data analysis over the past decades. Genomic or transcriptomic studies of aquatic animals under large-scale projects, such as Marine Mammal Genome project, Cetacean Genomes Project, Fish10K (The 10,000 Fish Genomes Project), and Fish-T1K (Transcriptomes of 1,000 Fishes), have greatly developed the knowledge in the field of evolutionary biology. Integration of genomics and transcriptomics provides efficient approaches to discover novel bioactive macromolecules from natural resources, such as antimicrobial peptides and toxins, and develop marine drugs based on them.

In this Research Topic, integrative genomic and transcriptomic data from various aquatic animals, including mollusks, crustaceans, amphibians, and fishes, provided valuable genetic resources for molecular insights into the omics-based investigation of bioactive products. It will help to develop a deeper understanding of the potential roles of diverse functional macromolecules and related networks. Getting started with this Research Topic, we hope to assemble an interesting edition that would highlight new developments and current trends in aquatic genomics and transcriptomics for study of differential gene expression and evolutionary selection, as well as for molecular characterization of bioactive macromolecules. The authors presented the current state-of-art in omics-based investigations and proved that aquatic organisms are invaluable resources of diverse bioactive compounds. The present Research Topic opens the gate for aquatic genomics and transcriptomics addressing adaptative molecular and physiological mechanisms as well as biology of diverse ecological systems.

Many bioactive molecules were identified in the immune, neuroendocrine, and gut systems of mollusks (Tascedda and Ottaviani, 2016). The worldwide scallops encompass various species of marine bivalve mollusks in the taxonomic family Pectinidae. The bay scallop *Argopecten irradians* is a commercially important bivalve mollusk cultured in the northern China (Ning et al., 2018). The purpose of genetic breeding in aquaculture is to obtain organisms with more genetic diversity for further selection of variants with superior economically important traits, such as rapid growth, disease resistance and high survival (Wang et al.). To broaden the genetic diversity of the bay scallops, artificial induction has been widely used to obtain strains with more mutations for further selection. In this Research Topic, Wang et al. applied the chemical mutagen ethylmethylsulfone (EMS) in the genetic breeding of scallops and characterized the mutations in growth-related genes. The results indicated the mutagenic effects of EMS by sequencing the genomes of 4 adult scallops from the control group and of 12 ones from the treatment group at 8 months after fertilization. On average, there were 1,151,380 ± 258,188 single nucleotide polymorphisms (SNPs) and 229,256 ± 51,714 insertion-deletions (InDels) in each animal in the EMS treatment group, while there were only 134841 ± 10,115 SNPs and 42,605 ± 5,136 InDels in the control group. The average mutation rate in the genome of the EMS-treated group (0.0137% ± 0.0013%) was about 9 times higher than that of the control group (0.0015% ± 0.0002%). Analyses of the growth-related genes with mutations indicated that mutations in Major Facilitator Superfamily (*MFS*) and Tubulin genes were found only in the large sized group, while mutations in Homeobox (*Hox*) and Suppressor of cytokine signaling (*Socs*) genes were revealed only in the smallest scallops. These results suggested that the above-mentioned genes might be involved in the regulation of scallop growth, wherein mutations in *MFS* and Tubulin genes may be related to fast growth in the large-sized group, while mutations in *Hox* and *Soc*s may contribute to the slow growth in the small-sized scallops. Besides, MS was shown to accelerate selection of economically important traits in molluscs. In this Research Topic, Wang et al. found that mutations in MFS and Tubulin may be involved in the fast growth of the large-sized scallops. However, those mutations in Homeobox and SOCs may be relevant to the slow growth in the small-sized group.

Crustaceans form a diverse arthropod taxon that contains about 67,000 described species including crabs, shrimps, lobsters, crayfish, prawns, krill, woodlice, and barnacles. Many crustaceans are free-living aquatic animals. The oriental river prawn *Macrobrachium nipponense* (Crustacea, Decapoda, Palaemonidae) is an important commercial species in China (Fu et al., 2012). The annual aquaculture production of this prawn has reached to 225,321 tons in 2019 (Zhang et al., 2020). Identification of genes, involved in the gonad differentiation, is important for implementation of the artificial technique to regulate the process of testis development in *M. nipponense*. In this Research Topic, Jin et al. aimed to determine the sensitive period of gonad differentiation and development using hematoxylin and eosin (HE) staining. Those important genes, involved in the gonad differentiation and development, were identified through transcriptome profiling analysis during the sensitive period of gonad differentiation and development. Based on the gene annotation and expression patterns, a total of 29 genes were identified as the candidate genes for potential involvement in the process of gonad differentiation and development in *M. nipponense* (Jin et al.). In this study, a total of 49,103 unigenes were assembled, and 18,430 of them were annotated. These genes were predicted to participate in cellular processes, signal transduction, posttranslational modification, protein turnover, and so on. This study not only provided a valuable evidence for establishment of the artificial technique to regulate the process of gonad development in *M. nipponense*, but also promoted the general understanding of crustacean evolution.

There are vast biological variety of species in four vertebrate classes including Amphibia (4,300), Reptilia (6,000), Aves (9,000), and Mammalia (8,000; West, 2018). Amphibians are one of the most diverse groups among vertebrates. The class Amphibia includes three orders: Gymnophiona (caecilians), Caudata (salamanders), and Anura (frogs and toads). *Anurans represent* the *most widespread* of the *three amphibian orders*: 25 families assembling over 4,000 species are currently recognized. Comprising a major clade of Anura, toads produce and secrete numerous toxins possessing pharmacological potential. However, the detailed genetic regulation of toad toxin production is still obscure. Recently, the genome of the representative Asiatic toad (*Bufo gargarizans*) was sequenced and annotated (Lu et al., 2021), providing a good opportunity to reveal the genetic differences between toads and frogs. Here, Lv et al. sequenced the transcriptomes of parotoid gland, dorsal skin, and liver from the Asiatic toad. These transcriptome datasets contained 51,668,570 reads (7,750,285,500 bp) in the parotoid gland, 38,894,474 reads (5,834,171,100 bp) in the dorsal skin, and 44,857,778 reads (6,728,666,700 bp) in the liver, respectively. Combining data obtained with 35 previously published transcriptomes across eight different tissues from the same species but from different locations, the authors constructed a comprehensive gene co-expression network of the Asiatic toad with assistance of the reference genome assembly. In summary, the authors organized a total of 38 transcriptomes covering eight different tissues for in-depth RNA analysis. A total of 2,701 co-expressed genes in the toxin-producing tissues (including parotoid gland and dorsal skin) have been identified. By comparative genomic analysis, the authors have identified 599 expanded gene families with 2,720 genes. Some of them, such as Cyp members, overlapped with the expanded genes and were significantly enriched into several important biosynthetic pathways, thereby illustrating the importance of these genes for the regulation of toad toxin production and secretion. Taken together, this study has uncovered transcriptomic and gene-family dynamic evidence of the vital role of both expanded gene copies and gene expression changes for production of toad toxins (Lv et al.).

The class “Pisces” includes more kinds of fish than all other vertebrates added together. Over 25,000 different species of fish are known, and new ones are discovered each year, so the final total number of fish species may exceed 30,000 (Bone and Moore, 2008). In this Research Topic, Tang et al. identified two types of digestive lipase genes pancreatic lipase (*pl*) and bile salt-activated lipase (*bsal*) in mammals and fishes. The neighborhood genes and key active sites of the two lipase genes were conserved in mammals and fishes. Three copies of *pl* genes were found in mammals, but only one copy of the *pl* gene was identified in most of the examined fish species, and the *pl* gene was absent in some fish species (e.g., zebrafish, medaka, and common carp). In contrast, the *bsal* gene existed in all examined fish species, but only one copy of the *bsal* gene was found in mammals, so the *bsal* might be the main digestive lipase gene in fish. The phylogenetic analysis indicated that the *pl* or *bsal* genes showed an independent evolution. By combining the phylogenetic results, Tang et al. inferred that the main digestive lipase gene evolved into two types between mammals and fishes. The pancreatic lipase might play an important role in lipids digestion in mammals and the PL occurred the gene tandem duplication. In contrast, the bile salt-activated lipase might play a major function in the lipid digestion in the fishes and the *bsal* occurred gene duplication in most of the fishes. The authors came to a temporary conclusion that the evolutionary selection of the main digestive lipase genes diverged into two types between mammals and fishes (Tang et al.). The genus *Prochilodus* includes 13 freshwater fish species living in rivers on both sides of the Andes mountains in Colombia, Venezuela, French Guiana, Suriname, Brazil, Peru, Bolivia, Argentina, Paraguay, and Uruguay (Castro and Vari, 2004). The Prochilodontidae (flannel-mouth characiforms) family comprises three phenotypically different genera including *Prochilodus*, *Semaprochilodus*, and *Ichthyoelephas*. Prochilodontids inhabit several river basins throughout South America and form massive populations of commercial fisheries (Melo et al., 2016). In this Research Topic, Yepes-Blandón et al. reported the first draft genome assembly for *Prochilodus magdalenae*, the leading representative species of the Prochilodontidae family in Colombia. Annotation identified 34,725 nuclear genes, and BUSCO completeness value was 94.9%. Gene ontology and primary metabolic pathway annotations indicate similar gene profiles for *P. magdalenae* and the closest species, blind cave fish (*Astyanax mexicanus*) and red piranha (*Pygocentrus nattereri*), with assembled genomes. A comparative analysis showed similar genome traits to other characid species. The fully sequenced and annotated mitochondrial genome reproduces the taxonomic classification of *P. magdalenae* and confirms the low mitochondrial genetic divergence inside the *Prochilodus* genus. Complete mitogenome phylogenies show that *P. vimboides* splits early from the common ancestor of the remaining *Prochilodus* species, followed by bocachico that splits form the ancestor shared by *P. lineatus, P. costatus, P. harttii*, and *P. argenteus*. Phylogenomic analysis, using nuclear single-copy orthologous genes, confirmed the evolutionary position of this species. Orthologous gene analysis confirmed that over 4,000 detected genes in the *P. magdalenae* annotation have respective orthologs in other *Actinopterygii* species, and single-copy orthologous genes showed a coherent phylogeny with a tree that reproduced the accepted topology for the species. Moreover, the *Characiformes* clade was segregated according to the classification of current higher rank group Otomorpha. This genome assembly provides a high-resolution genetic resource for sustainable management of *P. magdalenae* in Colombia and contributes to fish genomics throughout South America (Yepes-Blandón et al.). The tongue sole *Cynoglossus semilaevis* is a prevalent flatfish species in Chinese aquaculture due to its superior nutritive value and economic significance. Blind-side hypermelanosis is a major concern in commercial rearing environments of the flatfish aquaculture industry. In this Research Topic, Li et al. performed whole transcriptomic sequencing and analyses using normal and hypermelanic skins of the blind side of *C. semilaevis*. The authors identified differentially expressed long non-coding RNAs (DElncRNAs), miRNAs (DEmiRNAs), and differentially expressed genes (DEGs) as well as their competing endogenous RNA (ceRNA) networks. A total of 34 DElncRNAs, 226 DEmiRNAs, and 610 DEGs were identified. Finally, potential lncRNA–miRNA–mRNA regulatory networks (involving 29 DElncRNAs, 106 DEmiRNAs, and 162 DEGs) associated with blind-side hypermelanosis were constructed. This is the first study on the ceRNA regulatory network associated with blind-side hypermelanosis in flatfish (Li et al.). This investigation of functional roles of regulatory elements in the response to blind-side hypermelanosis might further contribute to selective breeding in flatfish. Melatonin works as the “gear” of the biological clocks in animals, which are influenced by light/dark or temperature changes in a timescale of day (circadian rhythms) or year (circannual rhythms) (Zhao et al., 2019). In fish, melatonin biosynthesis is regulated through the eyes and the pineal gland in response to light. Its biosynthesis is catalyzed by four cascaded enzymes including arylalkylamine N-acetyltransferase (AANAT). AANAT plays critical roles not only in melatonin biosynthesis, but also in dopamine metabolism responsible for seasonal migration, amphibious aerial vision, and cave or deep-sea adaptation. By contrast with vertebrates having only the single *aanat* gene, fish have several *aanat* genes (including *aanat1a, aanat1b,* and *aanat2*). With the rapid development of genome and transcriptome sequencing, more and more putative sequences of fish *aanat* genes becomes available. Related phylogeny and functional investigations will enrich our understanding of AANAT functions in various fish species. The review in this Research Topic summarizes the evolution and functions of AANAT in various fishes (Huang et al.).

Using whole-genome sequencing analysis in this Research Topic, Nedoluzhko et al. described clear and reliable signals of intergeneric introgression between the three-spined stickleback *Gasterosteus aculeatus* and its distant relative the nine-spined stickleback *Pungitius* that inhabit northwestern Russia. By contrast, the Japanese nine-spined stickleback specimen did not have a significant shift in allele frequencies compared with three-spined stickleback. The authors suggest that the moderate level of allele shift in the nine-spined stickleback population and the relatively low genetic distance between White Sea nine-spined stickleback and three-spined stickleback specimens are related to admixture events between these two species in this region of moderate salinity. By comparative analysis, the authors demonstrated that such introgression phenomena apparently took place in the moderate-salinity White Sea basin, although it was not detected in the Japanese sea stickleback populations. Bioinformatical analysis of the sites influenced by introgression showed that they were located near transposable elements, whereas those in protein-coding sequences were mostly detected in membrane associated and alternative splicing-related genes (Nedoluzhko et al.) The main result of this study is the discovery that the exchange of genetic material between distant taxa is much more common than previously expected. Data obtained by Nedoluzhko et al. demonstrate that in the moderate-salinity White Sea basin, three-spined stickleback found a more evolutionary distant mating partner.

In conclusion, the guest editors thank all authors who contributed to this Research Topic, all reviewers for evaluating the submitted manuscripts, and the editorial board members of *Frontiers in Genetics* for their continuous assistance to turn this Research Topic into reality.
